# An Algorithm for Retrieving Land Surface Temperatures Using VIIRS Data in Combination with Multi-Sensors

**DOI:** 10.3390/s141121385

**Published:** 2014-11-12

**Authors:** Lang Xia, Kebiao Mao, Ying Ma, Fen Zhao, Lipeng Jiang, Xinyi Shen, Zhihao Qin

**Affiliations:** 1 National Hulunber Grassland Ecosystem Observation and Research Station, Institute of Agricultural Resources and Regional Planning, Chinese Academy of Agricultural Sciences, Beijing 100081, China; E-Mails: xialang2012@163.com (L.X.); maying_helen@163.com (Y.M.); zhaofen198931@163.com (F.Z.); zhihaoqin@163.com (Z.Q.); 2 National Meteorological Information Center, China Meteorological Administration, Beijing 100081, China; E-Mail: jianglp@cma.gov.cn; 3 Hydrometeorology and Remote Sensing Laboratory, University of Oklahoma, Norman, OK 73072, USA; E-Mail: shen.xinyi@ou.edu

**Keywords:** land surface temperature, retrieval, MODIS, VIIRS

## Abstract

A practical algorithm was proposed to retrieve land surface temperature (LST) from Visible Infrared Imager Radiometer Suite (VIIRS) data in mid-latitude regions. The key parameter transmittance is generally computed from water vapor content, while water vapor channel is absent in VIIRS data. In order to overcome this shortcoming, the water vapor content was obtained from Moderate Resolution Imaging Spectroradiometer (MODIS) data in this study. The analyses on the estimation errors of vapor content and emissivity indicate that when the water vapor errors are within the range of ±0.5 g/cm^2^, the mean retrieval error of the present algorithm is 0.634 K; while the land surface emissivity errors range from −0.005 to +0.005, the mean retrieval error is less than 1.0 K. Validation with the standard atmospheric simulation shows the average LST retrieval error for the twenty-three land types is 0.734 K, with a standard deviation value of 0.575 K. The comparison between the ground station LST data indicates the retrieval mean accuracy is −0.395 K, and the standard deviation value is 1.490 K in the regions with vegetation and water cover. Besides, the retrieval results of the test data have also been compared with the results measured by the National Oceanic and Atmospheric Administration (NOAA) VIIRS LST products, and the results indicate that 82.63% of the difference values are within the range of −1 to 1 K, and 17.37% of the difference values are within the range of ±2 to ±1 K. In a conclusion, with the advantages of multi-sensors taken fully exploited, more accurate results can be achieved in the retrieval of land surface temperature.

## 1. Introduction

As the direct driver of the exchange of long-wave radiation and turbulent heat flux in the Earth-atmosphere system, the accurate measurements of land surface temperature (LST) are of great significance for monitoring global changes and crop conditions, specifically, climatic changes, drought monitoring, land surface evaporation monitoring, *etc.* [1,2]. Currently, data collected by weather stations and satellite remote sensing (RS) methods are generally the adopted methods for acquiring the LST data on a large scale. In contrast with the data collected by weather stations, the data acquired using RS methods can cover a huge observation range, which are slightly subjected to geographical restrictions. Moreover, non-point LST data can be obtained by RS methods. All of these merits make RS broadly applied in recent studies [3].

Acquisition of LST data from satellite data mainly includes the following two retrieval methods: thermal infrared (TIR) data and passive microwave data [4,5], respectively. Hardly affected by weather changes, the passive microwave data can reflect the LST around the clock; however, in terms of spatial resolution, the passive microwave data are far below the quality of data from the prevalent TIR sensors. To estimate the land surface temperature, the empirical, semi-empirical and physical retrieval models constructed by the passive microwave data exhibit comparatively poorer accuracy compared with the models established based on the thermal infrared data. Since first proposed by McMillin in 1975 [6], and after development for several decades, the algorithms for the retrieval of LST can be classified into single-channel, multi-channel and multi-angle methods. Using the single-channel method, the brightness temperature data from the single TIR channel are employed for the retrieval of LST on the conditions that the surface emissivity and atmospheric sections are known [7–11]. Qin *et al.* [9] have put forward a single-window algorithm applicable for the TM data. A generalized single-channel algorithm, advanced by Jiménez *et al.* [10,11] used a same inversion equation to retrieve the temperature data for different TIR sensors. By analyzing the retrieval accuracy of single-channel methods, Coll *et al.* [12] indicated that the accuracy could be enhanced effectively with the use of atmospheric profile products, such as National Centers for Environmental Prediction (NCEP) and the European Centre for Medium-Range Weather Forecasts (ECMWF). On the other side, the retrieval accuracy of different models could be greatly affected by the accuracy of atmospheric profiles, acquisition time and other factors [13].

Inspired by the split-window (SW) algorithm advanced by McMillin in 1975, which was originally used for the retrieval of ocean temperatures, many researchers have expanded the SW algorithm to the retrieval of land surface temperature [1]. Given that the atmospheric water vapor content and surface emissivity are known, Price has conducted studies on the LST retrieval based on the AVHRR data, with the use of SW algorithm [14]. Supposing that the atmospheric effects are invariable, Becker and Li have investigated the effects of surface emissivity on the retrieval accuracy [15]. Subsequently, the SW algorithm has been improved by Wan and Dozier [16], and the retrieval accuracy based on the data measured by the Advanced Very High Resolution Radiometer (AVHRR) and the Moderate Resolution Imaging Spectroradiometer (MODIS) have been enhanced with the observation angles of different sensors taken into account. Coll *et al.* [17] have established a novel retrieval algorithm, in which the zenith angle and surface emissivity observed by the corresponding sensors were added to the radiation transfer equation. A day/night temperature independent spectral indices (TISI) based method has been proposed by Li and Becker, in 1993, which could be applied to retrieve both the surface emissivity and temperature [18]. Some relevant improvements have also been made on the TISI-based method by Li *et al.*, Jiang *et al.* [19,20], Sobrino *et al.*, and Qin *et al.*, have performed studies on the retrieval of LST based on AVHRR data [21,22]. According to the research on the retrieval of LST using the MODIS data, a physics-based day/night operational method for simultaneous retrievals of temperature and emissivity, and a SW algorithm applicable to MODIS data have been proposed by Wan *et al.* [23–25]. In 2005, Mao *et al.*, have proposed a practical retrieval algorithm on the basis of the studies on the SW algorithm using the MODIS data [26]. Additionally, focusing on the Spinning Enhanced Visible and Infrared Imager (SEVIRI), Sun and Pinker have presented a four-channel retrieval algorithm, and performed the comparative analyses on the retrieval results between the proposed algorithm and various typical SW algorithms [27].

The multi-angle methods have been primarily designed for the Along Track Scanning Radiometer (ATSR). When observing at different angles, it can be found that the radiation emitted from the land surface presents different absorptions since the lengths of paths through the atmosphere are varying. The retrieval equations are then established in accordance with the different absorptions [28,29]. Sobrino and Jiménez-Muñoz have performed studies on the multi-angle and non-linear SW algorithms respectively, and the comparison results indicate that the multi-angle SW algorithm is of favorable retrieval accuracy [30]. Unfortunately, in practical applications, the multi-angle SW algorithm still has difficulties in achieving the accurate results, mainly due to the factors such as the dependency of emissivity on the observation angle, the significant effects of sensor noises on the accuracy, and the surface features at different viewing angles [28,31].

As stated above, where the history of the development of LST retrieval algorithms has been reviewed, we can conclude that the SW algorithms using TIR data are already very mature. Various SW algorithms dedicated to several kinds of data collected from MODIS [25], AVHRR [32], FY-2C [33], GOES-8 [34] and LandSat8 [35] have been studied. Different sensors exhibit different TIR spectral characteristics, and accordingly different retrieval equations. Besides, the acquirements of two key parameters, atmospheric transmittance and land surface emissivity, both of which are necessary for the construction of retrieval models, are different according to the varying spectral characteristics of different sensors. For instance, to perform the LST retrieval using AVHRR data, the atmospheric transmittance is generally set as a fixed value or calculated by the water vapor data obtained from the weather stations; while to retrieve the LST data using MODIS data, the water vapor data are measured by the MODIS sensors and thus the atmospheric transmittance is obtained. Obviously, with the adoption of the same retrieval algorithm, we can obtain more accurate water vapor data in satellite imaging by the measurement of sensors, compared with the point data measured by the land weather stations or the pre-set fixed values. Moreover, the measured water vapor data by sensors gain advantages in other aspects, such as the temporal synchronization and spatial distribution, contributing to a more desirable retrieval accuracy.

The visible infrared imager radiometer suite (VIIRS), carried by the Suomi National Polar-orbiting Partnership (Suomi NPP) polar-orbiting meteorological satellite which was launched on 28 October 2011, is referred to as the next-generation Earth observation instrument. By comparison with the MODIS data, the data collected by VIIRS exhibit significantly improved quality [36]. Due to the fact that no channel for the retrieval of water vapor is included in the VIIRS sensor, we should make efforts to seek for new methods to obtain the atmospheric transmittance, a key parameter in the construction of LST retrieval models. In the present work, a novel split-window (SW) algorithm has been developed based on the characteristics of data measured in mid-latitude region by the new sensor VIIRS. The problem of the absent water vapor channel in VIIRS have been solved in combination with the water vapor data obtained in MODIS. The derivation of the retrieval algorithm are described in Section 2, while the acquisition of water vapor data from MODIS and the effects of their estimation errors on the retrieval accuracy are analyzed in Section 3. In Section 4, we present the effects of the estimation error of soil emissivity on the total retrieval accuracy. Finally, the accuracy of the proposed algorithm was validated and a contrastive analysis was performed between the VIIRS retrieval data and the data measured by NOAA VIIRS LST products, which is discussed in Section 5.

## 2. Derivation of the Algorithm

The transmission of long-wave radiation is a fairly complex process, mainly including land surface radiation, atmospheric upward radiation and atmospheric downward radiation. After simplification by the derivation based on the physical models, the land surface radiation transmission in the TIR band [22,37] can be described as:

(1) $$B_{\lambda }\left(T_{\lambda }\right)=\;\tau_{\lambda }\left(\theta \right)[{ɛ}_{\lambda }B_{\lambda }\left(T_{s}\right)+\left(1-{ɛ}_{\lambda }\right)I_{\lambda }^{↓}]+I_{\lambda }^{↑}$$

in which *B_λ_*(*T_λ_*) denotes the radiance at the wavelength of *λ* received by the sensors at the top of the atmosphere (TOA); *λ* denotes the wavelength; *τ_λ_*(*θ*) denotes the transmittance of the land-emitted radiation passing through the atmosphere at the wavelength of *λ; ε_λ_* denotes the emissivity of the ground objects at the wavelength of *λ; B_λ_*(*T_S_*) denotes the radiation emitted by the ground objects;*T_S_* denotes the temperature of the ground objects; $I_{\lambda }^{↓}$ denotes the atmospheric downward radiation while $I_{\lambda }^{↑}$ denotes the atmospheric upward radiation. The calculations of the atmospheric upward and downward radiations in Equation (1) are comparatively complex. With reference to the studies by Qin *et al.* [22] and Mao *et al.* [26], another simplified equation to describe the transfer of land surface radiation can be expressed as:

(2) $$B_{\lambda }\left(T_{\lambda }\right)=\;\tau_{\lambda }(\theta ){ɛ}_{\lambda }B_{\lambda }\left(T_{s}\right)+(1-\tau_{\lambda }(\theta ))(1+\left(1-{ɛ}_{\lambda }\right)\tau_{\lambda }(\theta ))\;B_{\lambda }(T_{a})$$

in this equation, *T_a_* denotes the atmospheric upward radiation temperature. Consequently, with regard to the M15 and M16 channels in VIIRS, we can obtain the following expressions:

(3) $$B_{15}\left(T_{15}\right)=\;\tau_{15}(\theta ){ɛ}_{15}B_{15}\left(T_{s}\right)+(1-\tau_{15}(\theta ))(1+\left(1-{ɛ}_{15}\right)\tau_{15}(\theta ))\;B_{15}(T_{a})$$

(4) $$B_{16}\left(T_{16}\right)=\;\tau_{16}(\theta ){ɛ}_{16}B_{16}\left(T_{s}\right)+(1-\tau_{16}(\theta ))(1+\left(1-{ɛ}_{16}\right)\tau_{16}(\theta ))\;B_{16}(T_{a})$$

Items in Equations (3) and (4) can be expanded according to the Planck function [22], but this will increase the complexity of the equations. Former researchers [14,22] usually used a Taylor extension, or a linear regression method [26] to simplify Equations (3) and (4). In this article, a linear regression method is used. The temperature of M15 and M16 channels between 280 and 320 K are used to calculate the *B*_15_(*T*_15_) and *B*_16_(*T*_16_) by Planck function, and then the linear regression equation can be fitted between *B*_15_(*T*_15_)/*B*_16_(*T*_16_) and the temperature of M15/M16 channels:

(5) $$B_{15}\left(T_{15}\right)=0.1494T_{15}-34.934\;R^{2}=0.996$$

(6) $$B_{16}\left(T_{16}\right)=0.1239T_{16}-28.083\;R^{2}=0.997$$

in which *B*_15_(*T*_15_) and *B*_16_(*T*_16_) represent the radiance values of the M15 and M16 channels at the corresponding wavelength under the temperatures of *T*_15_ and *T*_16_, respectively; and *R*^2^ denotes the linearly dependent coefficient. To substitute the simplified Planck function into Equations (3) and (4), we perform a subtraction between two equations, aiming at eliminating of terms:

(7) $$T_{s}=a_{0}+a_{1}T_{15}+a_{2}T_{16}$$

in the equation, *a*_0_, *a*_1_ and *a*_2_ denote the coefficients, while *T*_15_ and *T*_16_ are the brightness temperatures of the M15 and M16 channels. *a*_0_, *a*_1_ and *a*_2_ can also be written as:

$$\begin{array}{c} a_{0}=\frac{C_{15}D_{16}-C_{16}D_{15}}{C_{16}A_{15}-C_{15}A_{16}}\\ a_{1}=\frac{0.1494C_{16}}{C_{16}A_{15}-C_{15}A_{16}}\\ a_{2}=\frac{-0.1239C_{15}}{C_{16}A_{15}-C_{15}A_{16}} \end{array}$$

where *A*_15_, *B*_15_, *C*_15_, *D*_15_, *A*_16_, *B*_16_, *C*_16_, and *D*_16_ are the equations related to the corresponding atmospheric transmissivity and land surface emissivity, with the expressions described as follows:

$$\begin{array}{c} A_{15}=\;0.1494\tau_{15}{ɛ}_{15}\\ B_{15}=\;0.1494T_{15}+34.934\tau_{15}{ɛ}_{15}-34.934\\ C_{15}=(1-\tau_{15})(1+(1-{ɛ}_{15})\tau_{15})\;0.1494\\ D_{15}=\;34.934\left(1-{ɛ}_{15}\right){\tau_{15}}^{2}\\ A_{16}=\;0.1239\tau_{16}{ɛ}_{16}\\ B_{16}=\;0.1239T_{16}+28.083\tau_{16}{ɛ}_{16}-28.083\\ C_{16}=\;(1-\tau_{16})(1+(1-{ɛ}_{16})\tau_{16})\;0.1239\\ D_{16}=\;28.083(1-{ɛ}_{16}){\tau_{16}}^{2} \end{array}$$

in which *τ*_15_ and *τ*_16_ are the atmospheric transmittance of M15 and M16 channels, respectively; *ε*_15_ and *ε*_16_ are the land surface emissivity of M15 and M16 channels, respectively. Conclusively, the measurements of atmospheric transmittance and land surface emissivity are necessary for the acquisition the LST data.

## 3. Acquisition of Water Vapor Data and the Related Sensitivity Analysis

### 3.1. Acquisition of Water Vapor Data and the Related Error Analysis

**Figure 1.**
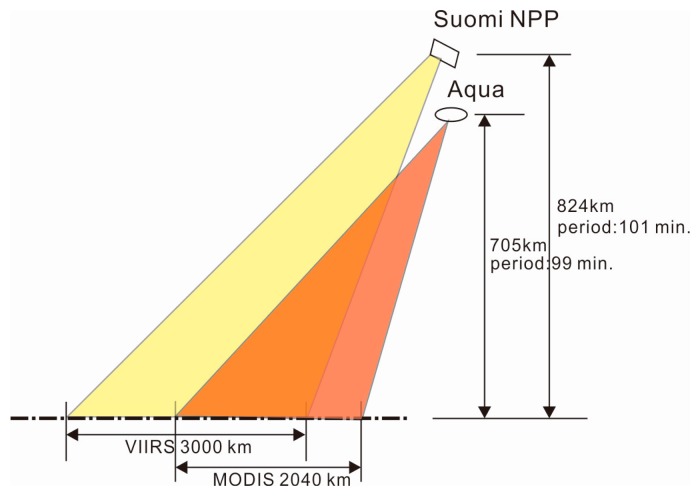
Part orbit parameters of the NPP and Aqua Satellites.

The atmospheric water vapor content is an important influencing factor on the atmospheric transmittance within the TIR wavelength range, and several vapor retrieval algorithms for MODIS [38], ATSR2 [39], and AATSR [40] have been proposed and verified with high accuracy. If the water vapor data cannot be directly measured by the satellite sensors, the water vapor data measured by the weather stations are generally adopted in the calculations of atmospheric transmittance when using the SW algorithm. However, the numbers of weather stations are limited, and the water vapor distribution may vary significantly among different regions. For these reasons, the selection of water vapor data from the weather stations will affect the accuracy of LST retrieval to a certain extent. Both the Suomi NPP satellite and the Aqua, a satellite in the National Aeronautics and Space Administration's (NASA) A-train constellation satellites, operate in a Sun-synchronous orbit with the ascending node of 13:30, so we can use the water vapor data collected by Aqua MODIS to remedy the lack of data which cannot be measured by NPP VIIRS. Figure 1 presents some orbital parameters of the NPP and Aqua satellites, respectively, from which we can observe that although they both move in a Sun-synchronous orbit with the ascending node of 13:30, the two satellites are different in terms of orbital altitude and the time to circle the Earth. Additionally, the swath of the VIIRS sensor, carried by the NPP satellite, is 3000 km, which is larger than that of MODIS, 2040 km. As a consequence, the acquisition of water vapor data by MODIS may exhibit two kinds of deviations, in which one is the time difference T observed in the same time zone induced by the different orbital periods, with the potential time difference ranging from 0 to 99 min; and the other deviation is induced by the different swaths between two sensors. Generally, two swaths of MODIS data are required to substitute for a swath of VIIRS data, and more seriously, three swaths of MODIS data are required in the worst cases. The resulting observation time difference is then less than or equal to 198 min, *i.e.*, T ≤ 198 min. In a conclusion, we should make a detailed analysis on the effects of the adoption of water vapor data from MODIS on the LST retrieval accuracy.

**Figure 2.**
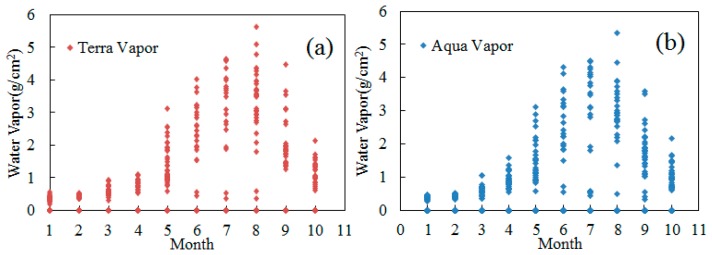
Comparison of the retrieved water vapor content from Aqua and Terra MODIS in Beijing area, January to August 2013.

The atmospheric water vapor contents in Beijing have been obtained by the retrieval of the Terra MODIS and Aqua MODIS data, with the scatter diagram of the data during the period from January to October in 2013 presented in Figure 2a,b. The points located on the x-axis represent the unsuccessful results for the retrieval of water vapor content because of the dense cloud coverage, whose value are 0. As shown in Figure 2, the atmospheric water vapor content varies with the seasonal changes. To be specific, from January to April, the water vapor contents are comparatively low, but the data points exhibit a centralized distribution. From May to August, owing to the effects of summer monsoon, the water vapor contents and their fluctuation increase, while a point-dense area can be observed in the scatter diagram in each month during this period; in July and August, with the increase of short-term convective weather in summer, the difference value of the water vapor content in one month can be as high as 4 g/cm^2^, which may impose significant effects on the retrieval accuracy of LST. According to the above-described analysis, in the present work, we perform an in-depth analysis on the conditions in August, the month exhibiting the greatest variations in the water vapor content.

**Figure 3.**
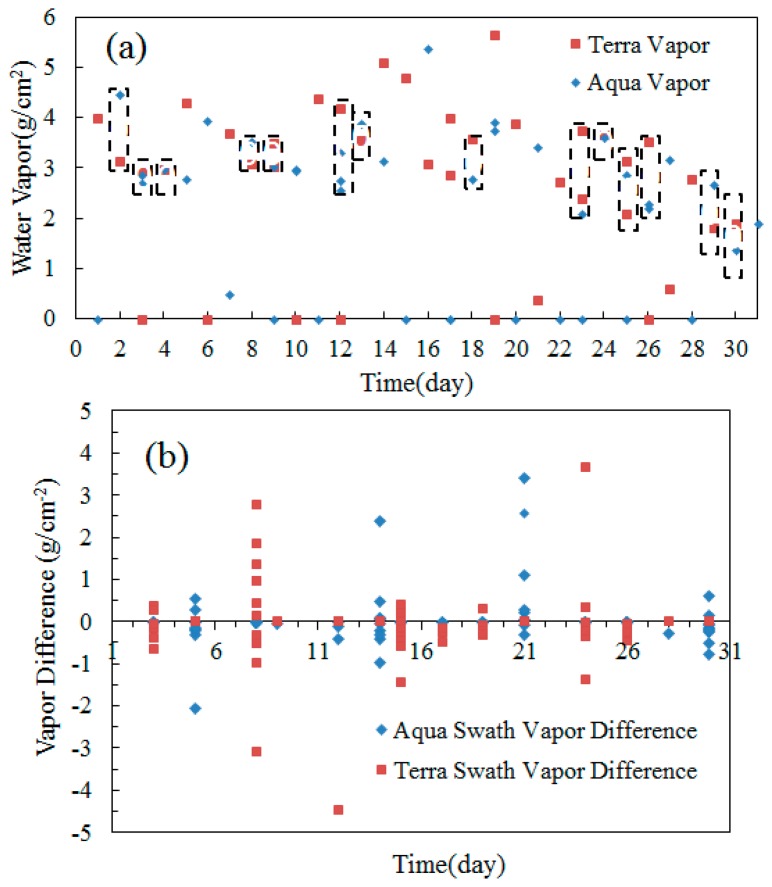
Comparison of the vapor retrieved from Aqua (afternoon) and Terra MODIS (morning) in Beijing area, August 2013 (**a**); Vapor retrieval difference between 2 and 3 swaths (at the same locations) measured by Aqua (afternoon) or Terra MODIS (morning) themselves in a larger area in August (**b**).

Figure 3a displays the retrieval results of the atmospheric water vapor content in Beijing in August, from the Terra MODIS data at 10:30 am and the Aqua MODIS data at 1:30 pm (1496 pixels are selected for each swath, and the vapor values shown in this figures are the average value of the 1496 pixels after removing the bad pixels). The dashed polygons in Figure 3a denote the days that are clear both in the morning and afternoon, while the polygons in the Figure 3a without dash boxes denote the days that are cloudy in the morning or afternoon (including thin cloud). Like Figure 2, the points located on the x-axis are the pixels contaminated by the clouds, whose values are 0. It can be observed from Figure 3a that, on sunny days, the difference values of water vapor contents measured in the morning and afternoon are relatively small, usually being less than 0.5 g/cm^2^.

Figure 3b displays the retrieval difference of vapor contents between 2 and 3 swaths (at the same locations) measured by Aqua (afternoon) or Terra MODIS (morning) in a larger area in August. The geographical coordinates of this area are N34 to 39°, E111 to 119°, and the vapor values obtain are obtained with the sampling interval of 1° in Figure 3b, the blue rhombi and brown squares denote the differences of water vapor contents measured within 2–3 orbital periods of Aqua and Terra MODIS, and the dashed line in Figure 3b refers to the vapor content of 0.5 g/cm^2^. As can be observed from Figure 3b, the vapor differences among most of the points are within 0.5 g/cm^2^ (the points with the difference values greater than 1.0 are mainly due to the weather change or cloud coverage). This means that the vapor changes in morning or afternoon on sunny days in this area are usually no greater than 0.5 g/cm^2^ within 2–3 orbital periods (about 198 min).

Table 1 lists the percentage of the vapor difference retrieved from Aqua and Terra MODIS in this area. As listed in Table 1, about half of the vapor difference values obtained in the morning and afternoon is within the range of 0.5 g/cm^2^. When the weather changes or the cloud coverage varies in the morning or afternoon, the vapor differences increase accordingly. In fact, under complicated weather conditions, the Aqua MODIS may fail to obtain the water vapor data, and the observation regions by Aqua MODIS are covered by clouds while the observation regions by NPP VIIRS may be clear. In these cases, the water vapor data observed by Terra MODIS are employed, and the estimation errors may be equal to or greater than 2.0 g/cm^2^. Conclusively, when using the water vapor data observed by Aqua MODIS for LST retrieval, the estimation errors on sunny days are no more than 0.5 g/cm^2^ while the errors under complicated weather conditions are approximately 2.0 g/cm^2^ (in fact, the uncertainties for the acquisition of water vapor data are even more serious when the weather conditions are complicated).

### 3.2. Calculations of Atmospheric Transmittance and Sensitivity Analysis

#### 3.2.1. Calculations of Atmospheric Transmittance

**Figure 4.**
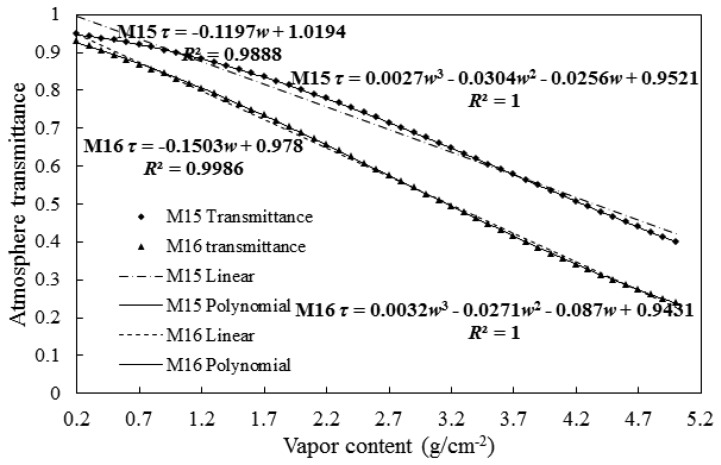
The relationships between the water vapor content (*w*) and atmospheric transmittance (*τ*) for the mid-latitude regions in summer, with the data collected by the M15 and M16 channels of VIIRS.

When the water vapor content varies in a small range, it presents a remarkable correlation with the atmospheric transmittance. Sobrino *et al.* and Qin *et al.* have adopted a linear equation to perform simulations on the data of water vapor content and transmittance, in which the water vapor data were collected by channels 4 and 5 of AVHRR [21,22]. Using the data observed by channels 31 and 32 of MODIS, Mao *et al.*, have studied the relationship between the water vapor content and transmittance by means of an exponential equation [26]. In the present work, the atmospheric transmittance corresponding to the different water vapor contents were simulated for the mid-latitude regions in summer by MODTRAN software, with the data observed by the M15 and M16 channels of VIIRS. The correlations between the water vapor content and atmospheric transmittance were studied by linear fitting and polynomial fitting, respectively, with the results displayed in Figure 4. Whether fitted by a linear equation or a polynomial, favorable correlations between them can be observed. However, when fitted by a linear equation, the results for some water vapor contents for the data collected by the M15 channel are relatively poorer, such as the conditions that the water vapor content is below 1.0 g/cm^2^, within the range from 1.2 to 2.2 g/cm^2^ and above 4.3 g/cm^2^. For the data collected by the M15 and M16 channels, the correlations between the water vapor content and atmospheric transmittance can be fitted more accurately by polynomials, with the fitting coefficients of 1. Accordingly, the formula adopted for the calculation of atmospheric transmittance in the present work can be expressed as (w is the vapor content, g/cm^2^):

(8) $$\tau_{15}=0.0027w^{3}-0.0304w^{2}-0.0256w+0.9521$$

(9) $$\tau_{16}=0.0032w^{3}-0.0271w^{2}-0.087w+0.9431$$

#### 3.2.2. Sensitivity Analysis

The errors regarding the retrieval accuracy of the present models are analyzed and listed in Table 2. The mid-latitude regions in summer with the land surface covered by vegetation, water, sand, city, soil were adopted (the emissivity of the M15 and M15 bands can be found at Section 4.1, and all the emissivity used in this article are the band-averaged emissivity of the specific land type), and the estimation errors of water vapor content ranged from −1.5 to 1.5 g/cm^2^. The atmospheric transmittance was calculated by inputting the water vapor data into the radiation transfer software MODTRAN. As listed in Table 2, *T_t_* denotes the inputted LST parameter in MODTRAN; Δ*T*_s_ denote the retrieval errors induced by the estimation errors of water vapor content, when the water vapor content is 2.5 g/cm^2^ and the estimation errors are ±0.5, ±1.0 and ±1.5 g/cm^2^, respectively. Δ*T*_s_ is calculated in the following equation:

(10) $$\Delta T_{\text{s}}=\left|\left|T_{t}-T_{s}\right|-\left|T_{t}-T_{sv}\right|\right|+\left|T_{t}-T_{sv}\right|$$

where *T_sv_* denotes the retrieval result of LST with the vapor content of 2.5 ± 0.5, 2.5 ± 1.0 and 2.5 ± 1.5 g/cm^2^, respectively, while *T_s_* is the retrieval result of LST with the base vapor content of 2.5 g/cm^2^.

As is shown in Table 2, for each land type, the LST estimate errors increase with the increase of vapor estimate errors. The backgrounds of vegetation and water exhibit the larger LST errors than other backgrounds, when the estimation errors of vapor are within the range of −1.5 to −0.5 g/cm^2^. In general, when the estimation errors of water vapor content is approximately ± 0.5 g/cm^2^, the mean LST estimation error of the algorithm for five land types is 0.634 K, and the mean standard deviation value is 0.245 K. The maximum mean LST estimation error for five land types is 0.736 K, the mean standard deviation value is 0.280 K while the water vapor content is ± 1.5 g/cm^2^. Therefore, on sunny days, the LST retrieval errors of the algorithm caused by vapor estimation errors are no greater than 0.634 K.

## 4. Estimation of Land Surface Emissivity and Sensitivity Analysis

### 4.1. Estimation of Land Surface Emissivity

It is well-documented that the land surface emissivity is one of the key parameters in the retrieval of LST. There have been a lot of detailed studies on the land surface emissivity corresponding to the related bands of sensors, including AVHRR, MODIS, ASTES, *etc.* [18,19,41,42] In these previous studies, the researchers often used a pre-determined emissivity value, or obtained the emissivity value for the same type of land surface according to the classification data. Besides, the emissivity of the mixed pixel can be calculated based on the ratio of the Normalized Difference Vegetation Index (NDVI) to soil at the pixel. If a pre-determined emissivity is adopted, we should know the pixel emissivity in advance. Similarly, when performing the land surface classification-based algorithm, the prior knowledge of the emissivity for each type of land surface should be provided. Furthermore, the land surface emissivity also varies with the seasonal changes. The mixed-pixel based calculation method can be only applied for the pixel regions containing soils and vegetation; however, for the regions covered by water, rock, ice and snow, the method may be unsuitable [1]. In the present work, the data of land surface emissivity were identified by combining the land classification based method with the mixed pixel method to avoid limitations mentioned previously. For some regions whose surface are almost unchanged, such as the forest, the regions with high vegetation coverage, soil (NDVI < 0.1), water, desert and the urban built-up areas, the adopted emissivity values are listed as follows (the data sources: ASTER spectrum library and VIIRS LST ATBD):

$$\begin{array}{c} \text{Vegetation}:\;{ɛ}_{15}=0.990\;{ɛ}_{16}=0.990\\ \text{Soil }\left(\text{dry}\right):\;{ɛ}_{15}=0.963\;{ɛ}_{16}=0.974\\ \text{Soil }\left(\text{wet}\right):\;{ɛ}_{15}=0.979\;{ɛ}_{16}=0.974\\ \text{Water}:\;{ɛ}_{15}=0.990\;{ɛ}_{16}=0.990\\ \text{Desert}:\;{ɛ}_{15}=0.963\;{ɛ}_{16}=0.985\\ \text{City}:\;{ɛ}_{15}=0.974\;{ɛ}_{16}=0.979 \end{array}$$

On the other hand, for the crop land whose coverage varies with the different planting system and seasons, the emissivity can be estimated by the mixed pixel method if its NDVI is within the range from 0.1 to 0.65, as listed in the following expressions:

(11) $${ɛ}_{15}=0.963\times \left(1-P_{v}\right)+0.990\times P_{v}$$

(12) $${ɛ}_{16}=0.974\times \left(1-P_{v}\right)+0.990\times P_{v}$$

in which *ε*_15_ and *ε*_16_ are the emissivity of the corresponding pixels of M15 and M16, respectively; and *P_v_* denotes the ratio of vegetation in the pixel and can be calculated as:

(13) $$P_{v}=(\text{NDVI}-{\text{NDVI}}_{s})/({\text{NDVI}}_{v}-{\text{NDVI}}_{s})$$

where *NDVI_s_* = 0.05, *NDVI_v_* = 0.65 (generally, the region whose NDVI is less than 0.05 is referred as to bare land while the region whose NDVI exceeds 0.65 represents the regions totally covered by vegetation) [43].

### 4.2. Sensitivity Analysis

**Figure 5.**
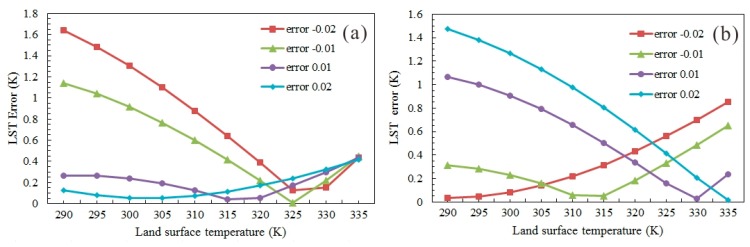
Probable LST estimate errors due to emissivity estimate errors (±0.02) in VIIRS channel M15 (**a**) and M16 (**b**).

**Figure 6.**
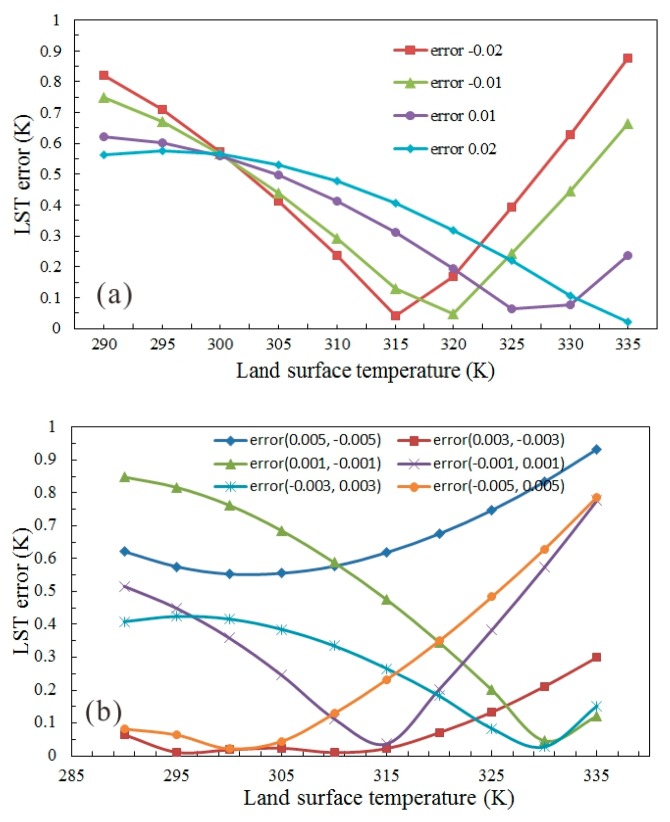
Probable LST estimate errors in both VIIRS channel M15 and M16 with same emissivity estimate errors ±0.02 (**a**) and (0.005, −0.005), (0.003,−0.003), (0.001,−0.001), (−0.001, 0.001), (−0.003, 0.003), (−0.005, 0.005) for each (**b**).

The present retrieval algorithm have been executed when the estimation error of land emissivity by VIIRS was less than or equal to ±0.02, with a varying LST temperature from 290 to 335 K. Figure 5 displays the retrieval errors of LST using the data from the M15 and M16 channels (dry soil), with the emissivity of 0.963 and 0.974, respectively. As shown in Figure 5a, for the data collected by the M15 channel, when the estimation error is negative, the LST retrieval error reduces significantly with the increase of LST temperature; when the estimation error is positive, the retrieval error is slightly affected by the variation of temperature. The results for the data collected by the M16 channel, as shown in Figure 5b, present a completely different variation tendency. Overall, for the data collected by the two channels, more desirable retrieval accuracy can be obtained at lower LST temperatures. When the estimation errors of emissivity were within the range of ±0.02, their effects on the retrieval accuracy were studied, with the results displayed in Figure 6a. For the data collected by the two channels, the retrieval accuracy of the present algorithm is less than 1 K even the estimation error of emissivity is as high as ±0.02. The retrieval errors induced by the same estimation errors of emissivity are opposite between the results from the M15 and M16 channels, which contributes to the improvement of algorithm accuracy on the whole.

Regarding the sensitivity of emissivity estimation errors by the AVHRR and MODIS data on the LST retrieval, a large number of achievements have been obtained by previous researchers. In the LST retrieval studies for the AVHRR data performed by Qin *et al.* [22], the error of the adopted algorithm is 0.708 K when the estimation error of emissivity is 0.01. In the studies for the MODIS data by Mao *et al.* [26], the retrieval error is 1.116 K when the emissivity estimation error is 0.02. As can be observed, in the present algorithm, the retrieval error is less than 0.820 K when the emissivity error is 0.02 (the LST is 335 K and the water vapor content is 1.8 g/cm^2^). The result suggests that, compared with the retrieval using the AVHRR and MODIS data, the LST retrieval using the data collected by the M15 and M16 channels of VIIRS exhibits favorable tolerance with the same emissivity estimation errors.

Estimation errors in the opposite direction of emissivity in M15 and M16 channels are performed to make an in-depth complete analysis. If the estimation errors are ±0.02, the results may be unrealistic, for example, if the emissivity of M15 and M16 channels are 0.943 and 0.994 with an estimation error of (−0.02,0.02), then it is not consistent with the emissivity of the real material. According to the former studies, the uncertainty of emissivity is around 0.005 in the opposite direction in 11 μm and 12 μm bands [16]. In this part, the emissivity estimation errors of (0.005, −0.005), (0.003, −0.003), (0.001, −0.001), (−0.001, 0.001), (−0.003,0.003), (−0.005, 0.005) are used for M15 and M16 to conduct the simulation analysis, as shown in Figure 6b. In Figure 6b, the maximum LST errors are within 1 K with the different emissivity errors. Consequently, the LST estimation error is less than 1.0 K when the M15 and M16 channels emissivity estimation errors in the opposite direction is within −0.005 to +0.005 K.

## 5. Validation of the Accuracy and the Application Analysis

Accuracy validation is one of the important steps in the construction of a retrieval algorithm. Without any relevant validations, the proposed retrieval algorithm cannot be used for the acquisition of various parameters of RS with certainty. Currently, in regard to the validation of LST retrieval algorithm, the temperature-based method, the radiance-based (R-based) method and the combined methods are often used [1,44,45]. In this paper, the R-based method was employed for assessing the accuracy of the proposed algorithm, and then the actual data from VIIRS were compared with the LST measured by the ground stations (temperature-based method) and the NOAA LST measurement products for comparative evaluations.

### 5.1. Validations by the Standard Atmospheric Simulation

The radiance-based method is a preferable validation method to substitute for the temperature-based method [1,21,22,27]. By inputting the land surface emissivity, the atmospheric profile status and the retrieval LST, the TOA radiance can be simulated by the radiation transfer equation and then the retrieval accuracy can be assessed. The mid-latitude regions in summer and winter were selected on sunny days to validate the accuracy of the algorithm, with a varying temperature from 290 to 320 K. In the construction of retrieval models, several types of underlying surface were adopted, including soil, vegetation, city, desert and water (it should be pointed out that the soil type used in summer is wet while the soil used in winter is dry). Accordingly, the water vapor content was varied, to be 0.8, 2.3 and 3.8 g/cm^2^, respectively. The retrieval accuracy have been simulated and analyzed by MODTRAN software, with the results listed in Table 3. For the above-mentioned five types of land surface, the mean retrieval error is 0.570 K and the mean standard deviation value is 0.331 K.

Since the accuracy analysis was calculated in summer and winter for the mid-latitude regions, some input parameters that could not fit with the summer or winter characteristics would generate large errors. As shown in Table 3, for the soil-type (wet soil) land surface, the retrieval error using the present algorithm is fairly high at the LST of 290–320 K, which exceeds 1 K when the water vapor content is 0.8 g/cm^2^. In practice, it is nearly impossible for the wet soil to have a high LST of 320 K and a low water vapor of 0.8 g/cm^2^. This leads to the inconformity between the retrieval results and the actual results. Similarly, for the city-type (winter) land surface when the LST temperature is 320 K and the water vapor content is 3.8 g/cm^2^, the retrieval errors are still significant. The same case can also been observed for the water-type land surface, with the LST and water vapor content being 320 K and 0.8 g/cm^2^, respectively.

Additionally, 23 surface types (the information of the surface types can be found at NOAA VIIRS LST algorithm [46]) were used to validate the accuracy of the algorithm adopted in both summer and winter, with a varying temperature from 280 to 320 K and vapor from 0.4 to 3.9 g/cm^2^ (to be concise, the results are not shown at full length in Table 3). The mean retrieval error is 0.734 K and the mean standard deviation value is 0.575 K, which indicates that retrieval error is less than 1 K. In conclusion, the LST data with high accuracy can be obtained by VIIRS with the adoption of the present algorithm.

### 5.2. Validations by the Data from Ground Stations

#### 5.2.1. The Introduction of the Ground Stations Data and Processing Rules

The ground stations data were provided by China Meteorological Data Sharing Service System, and the data were measured with the ground meteorological observation standard [47]. The ground station LST data were measured hourly, and if the ground station meets the following conditions, it would be removed: the ground station was located in area with mixed land coverage, such as water and vegetation; the time difference between data acquisition of the sensor and the ground station was greater than one minute; the ground station was covered by cloud; for crop land, the NDVI was less than 0.5. Besides, if the scan angle of the sensor is greater than 32°, then the ground station under those pixels would not be used to validate the result. This was mainly because the resolution of the VIIRS data increased with the increases of scan angle, for example, when the scan angle is 32°, the resolution will be greater than 1 km (VIIRS nadir resolution is 0.75 km), which may cause the results more uncertainty.

#### 5.2.2. Results

**Figure 7.**
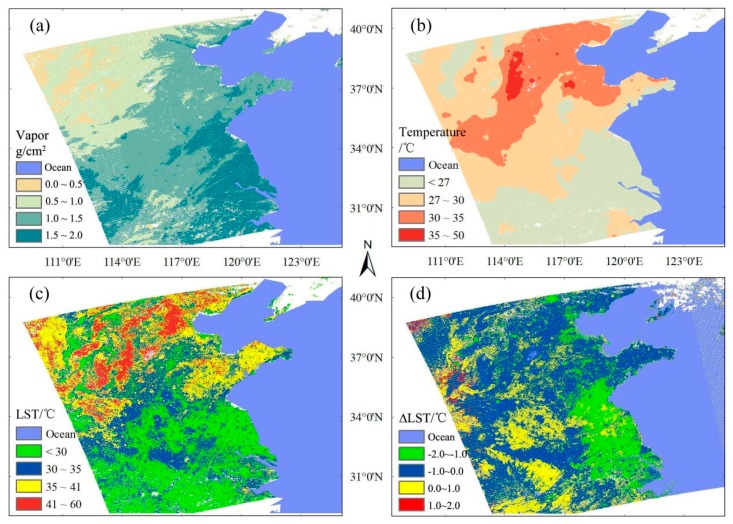
The retrieve result of this study. (**a**) VIIRS vapor data get from MODIS vapor product MYD05_L2; (**b**) Temperature of air (2 m height); (**c**) LST retrieval LST of this study; (**d**) LST difference between the NOAA official and this study.

The retrieval results of LST with the true VIIRS data are displayed in Figure 7, in which the VIIRS data were imaged at 05:01 UTC, on 11 May 2013. Figure 7a also presents the obtained water vapor data by the Aqua MODIS data, at 04:55 UTC, on 11st May 2013; Figure 7b presents the atmospheric temperature data at a distance of 2 m away from the land surface, which are obtained by the International Meteorological Swap Data Center, at 05:00 UTC, on 11st May 2013; while Figure 7c presents the retrieval results of LST. The related underlying land surface in the present work includes crop land, city, the built-up areas and water. As shown in Figure 7a, the water vapor content varies significantly among the different regions; specifically, the water vapor content in the low-latitude and coastal regions are comparatively higher while the content in the inland regions are lower. The water vapor data obtained by Aqua MODIS can effectively remedy the absent of water vapor data by VIIRS. As we all know, the atmospheric temperatures near the land in Figure 7b are not equal to the LST data in Figure 7c, however, it reflects temperature distribution tendencies are consistent. As shown in Figure 7b, the atmospheric temperatures near the land surface are higher in northern China than those in southern China, which is in good consistency with the LST distribution as described in Figure 7c.

Using the temperature-based validations method, the related LST parameters of the pixel are obtained and compared with the results measured by the ground stations (when the satellite passes). Unfortunately, in actual situations, the compositions of land surface are quite complicated. The land surface corresponding to a single pixel often occupies a large area (VIIRS 0.562 5 km^2^), and often pixels are mixed. Besides, some uncertainties exist in the scale transformation between the ground station data and satellite data. All of these lead to the restrictions of the applications of temperature-based method in practical. To overcome this difficulty, we often select the retrieval results for the regions with an even land surface for comparative validations. In the present study, the VIIRS data were measured in the middle of May, during the growing period of wheat. With a large NDVI, the regions exhibited an even underlying surface, and therefore are suitable for the comparative analysis on the accuracy (as shown in Table 4, the data were collected at 05:00 UTC, on 11 May 2013). Similarly, the measured region for the water is West Lake, covering an area of 5.6 km^2^, which is also characterized by an even surface. The land surface of city is quite complicated, so the validation of LST retrieval accuracy for city is much more difficult than the accuracy validation for the regions covered by even vegetation and water. Some related data for the city regions are listed in Table 4 for reference only. It can be concluded from Table 4 that, in water regions, the retrieval results fit well with the data from the ground stations; while the regional fluctuations in crop land and city are comparatively large, but the differences value is less than 1.5 K, suggesting that the retrieval results accord well with the data measured by the ground stations.

**Figure 8.**
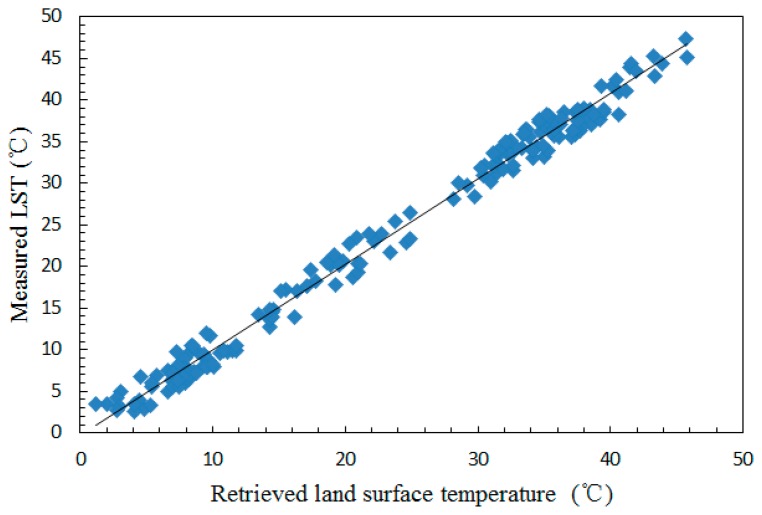
Validation of the algorithm using difference VIIRS data and the ground stations data.

Due to the cloud coverage, the difference between data acquisition time of the sensors and the ground stations, the scale difference between the ground station (point) and pixel (surface), verifying the accuracy of the algorithm bases on a long time series is a very difficult task. On the other hand, the ground station data used in the validation were measured by hour, which makes the validation of the algorithm more difficult. In this section, twelve VIIRS swaths were selected to validate the algorithm, and this data were collected ranging from May 2013 to June 2014 (most of this data are in winter and summer), as shown in Figure 8 (the data shown in Table 4 also be used in the figure). The transmittance equations adopted for the calculation of atmospheric transmittance in winter and summer were different, and for mid-latitude winter, the equations were as follows:

(14) $$\tau_{15}=0.0027w^{3}-0.0304w^{2}-0.0255w+0.9524$$

(15) $$\tau_{16}=0.0032w^{3}-0.0271w^{2}-0.087w+0.9434$$

A match line in the figure indicates the retrieved and measured LST are similar, and the mean retrieval error and the standard deviation value of mean error shown in Figure 8 are −0.395 K, and 1.490 K, respectively. These results indicate the adopted LST algorithm is appropriate.

### 5.3. Comparative Analysis with NOAA LST Product

The NOAA VIIRS LST algorithm consists of two types of algorithm, the split algorithm (SW) and dual-split window (DSW), and all the coefficients in each equation are obtained by regression; detailed derivation of the algorithm can be found at [46]. The retrieval results of LST compared with the results from the LST measurement products of NOAA (the DSW algorithm are adopted before 10 August 2012) [48], is shown in Figure 7d. The regions colored in blue, occupied the 54.04% of the whole pixels, denote the pixels with the difference values of LST ranging from −1.0 to 0.0 K; the yellow regions with difference values ranging from 0.0 to 1.0 K occupy 28.59%; while the green and red regions, with difference values ranging from −2.0 to 1.0 K and from 1.0 to 2.0 K, respectively, take up 13.98% and 3.39% of the whole pixels. Accordingly, the pixels with the difference values ranging between −1.0 to 1.0 K occupy 82.63% while the pixels with the difference values larger than 1 K occupy 17.67%. The regions with larger difference values are mainly city, water and some crop lands, and we have evaluated the retrieval accuracy of the present algorithm using the R-based method.

When the underlying land surface are characterized by water, city and crop lands, the simulated LST data by the MODTRAN software in the present work are compared with the data obtained by the LST measurement products of NOAA, with the results listed in Table 5. The data of land surface were selected from Figure 7, and the detailed parameters can be consulted in Table 6. In the simulations by MODTRAN software, with the assumption that the water vapor data and emissivity are pre-defined, the brightness temperatures of the M15 and M15 channels of VIIRS were simulated by inputting the retrieval results of LST. The simulation results were compared with the actual results, as shown in Table 6. The less the difference values between the simulation results and the actual results, the less the retrieval errors. In Table 5, the emissivity *ε*_15_ and *ε*_16_ in the row of ‘Our’ are the emissivity corresponding to the crop land surface in NOAA VIIRS ATBD; the emissivity in the bracket is the emissivity of this kind of land surface in the present study and the LST denotes the retrieval results of land surface temperature corresponding to the emissivity.

As shown in Table 6, Crop1_0.68_ and Crop2_0.68_ denotes the simulation results of the brightness temperatures of the M15 and M16 channels with the use of the emissivity data from NOAA VIIRS ATBD and the present calculations, respectively; for the vegetation with a NDVI of 0.68. Similarly, Crop1_0.30_ and Crop2_0.30_ are the brightness temperatures by performing simulations on the crops with a NVDI of 0.30. Δ_M15_ and Δ_M16_ are the difference values of the brightness temperatures between the simulation channels and the satellite. Mean 1 Error and Mean 2 Error denote the mean errors of the difference values of the brightness temperatures between the simulation channels and the satellite, simulated by the emissivity data from the VIIRS ATBD and the calculated results in the present work, respectively.

As can be concluded from the results in the rows of Δ_M15_ and Δ_M16_, for the water-type and city-type land surface, the retrieval errors calculated by the present algorithm are less than these of NOAA LST products. For the crop land, when using the emissivity obtained by the VIIRS ATBD, the mean retrieval errors by the present algorithm and the official algorithm of NOAA are larger than the errors using the emissivity data calculated by the present algorithm. The results indicate that, for the crop land, the adoption of the pre-defined values of land emissivity have several limitations. The variations of emissivity induced by the variations of planting system and seasons cannot be taken into the consideration of retrievals immediately, producing large errors. Comparatively, the present algorithm, in which the crop land surface emissivity is estimated with the adoption of the mixed pixel method, can provide high-accuracy results.

## 6. Conclusions

In the present work, according to the data characteristics of the novel sensor VIIRS, we constructed a modified SW algorithm for mid-latitude regions, combined with multi-sensor data to obtain water vapor content. The related sensitivity analyses were performed by MODTRAN software, and the results were compared with the results measured by the NOAA LST products, with the main conclusions listed as follows:

- The water vapor content obtained by the Aqua MODIS can effectively remedy the absent of water vapor data measured by VIIRS. The results indicate that the maximum estimation error of water vapor content is 0.5 g/cm^2^ on sunny days while the estimation of water vapor content exhibits a comparatively large uncertainty under complicated weather conditions, to be approximately 2.0 g/cm^2^ (the uncertainties for the acquisition of water vapor data are more serious under complicated when the weather conditions are complicated).
- By analysis on the simulation data, we can conclude that the estimation errors of land surface by the VIIRS data provide preferable tolerance compared with the errors by the AVHRR and MODIS data. Accuracy analysis in the study shows, the average LST retrieval error for the twenty-three types of land types is 0.734 K, with a standard deviation value of 0.575 K. By comparisons with the data measured by ground stations, the difference values between the retrieval results in the present work and the measured data measured are less than −0.395 K (mean standard deviation error 1.490 K). Besides, the retrieval results of the test data were compared with the results obtained by the NOAA LST products, indicates that 82.63% of the difference values are with the range from −1.0 to 1.0 K, the retrieval results by these two methods are quite close with each other, especially in terms of accuracy. As can also be concluded from the further studies, the regions with large difference values between the retrieval results and the data measured by NOAA LST products are generally the crop land, whose emissivity are significantly affected by the variations of planting system and seasons. Comparatively, for these regions, the present algorithm in which the land surface emissivity is estimated using the mixed pixel method can provide high-accuracy results. Conclusively, with the advantages of multi-sensors taken fully exploited, more accurate results can be achieved in the retrieval of earth observation parameters.

## Figures and Tables

**Table 1.** Percentage of the difference of the vapor retrieved by Aqua (afternoon) and Terra MODIS (morning) in the same area, in August 2013.

**Vapor Difference (g/cm^2^)**	**>6.0**	**6.0–5.0**	**5.0–4.0**	**4.0–3.0**	**3.0–2.0**	**2.0–1.0**	**1.0–0.5**	**0.5–0.0**
Percentage	0.109	0.434	2.172	3.040	6.406	17.590	20.521	49.729

**Table 2.** Probable LST estimate errors with the vapor retrieve errors.

**Land Type**	***T_t_*/K**	**Δ*T*_s_/*K* Vapor Error −1.5∼−0.5 g/cm^2^**			**Δ*T*_s_/*K***	**Δ*T*_s_/*K* Vapor Error 0.5∼1.5 g/cm^2^**		
		**–1.5**	**–1.0**	**–0.5**	**0.0(2.5 g/cm^2^)**	**0.5**	**1.0**	**1.5**
Vegetation	290	1.061	0.914	0.831	0.698	0.698	0.698	0.698
	300	1.017	0.916	0.872	0.760	0.760	0.760	0.760
	310	1.023	0.921	0.856	0.678	0.678	0.678	0.678
	320	1.057	0.922	0.788	0.485	0.485	0.485	0.485

Water	290	1.078	0.870	0.684	0.448	0.448	0.448	0.448
	300	1.134	0.957	0.800	0.573	0.573	0.573	0.573
	310	1.234	1.047	0.859	0.555	0.555	0.555	0.555
	320	1.359	1.131	0.863	0.424	0.424	0.424	0.424

Soil	290	0.810	0.714	0.698	0.666	0.666	0.666	0.666
	300	0.572	0.570	0.570	0.570	0.570	0.570	0.570
	310	0.386	0.354	0.378	0.330	0.330	0.330	0.330
	320	0.240	0.185	0.142	-0.028	0.085	0.463	1.145

Sand	290	1.088	1.088	1.088	1.088	1.088	1.103	1.088
	300	0.947	0.947	0.947	0.947	0.947	0.979	0.947
	310	0.663	0.663	0.663	0.663	0.663	0.663	0.663
	320	0.262	0.262	0.262	0.262	0.262	0.262	0.262

City	290	0.930	0.849	0.839	0.808	0.808	0.808	0.808
	300	0.742	0.742	0.742	0.742	0.742	0.742	0.742
	310	0.565	0.548	0.577	0.532	0.532	0.532	0.532
	320	0.448	0.410	0.372	0.204	0.204	0.204	0.452

Mean error		0.831	0.751	0.691	0.570	0.576	0.597	0.641

STD error		0.328	0.279	0.243	0.258	0.246	0.223	0.232

Average:								

**Table 3.** Verify the accuracy of the study with MODTRAN. *T_s_* denotes the retrieval result of LST, *T_t_* denotes the inputted LST parameter in MODTRAN. *T_s_* – *T_t_* is the retrieval errors of the algorithm.

**Atmospheric Model**	**Water Vapor g/cm^2^**		**Transmittance**		***T_t_*/K**	**Land Type |*T_s_* – *T_t_*|/K**				
	
			**M15**	**M16**		**Soil**	**Veg**	**City**	**Sand**	**Water**
mid-latitudes summer	0.8		0.913	0.856	290	**1.179**	0.946	0.917	1.006	0.829
	0.8		0.913	0.856	300	**1.257**	0.952	0.772	0.761	0.918
	0.8		0.913	0.856	310	**1.295**	0.915	0.588	0.478	0.963
	0.8		0.913	0.856	320	**1.298**	0.848	0.373	0.167	0.976
	2.3		0.766	0.640	290	0.760	0.728	0.783	1.070	0.499
	2.3		0.766	0.640	300	0.878	0.790	0.719	0.922	0.626
	2.3		0.766	0.640	310	0.872	0.726	0.480	0.647	0.627
	2.3		0.766	0.640	320	0.764	0.562	0.103	0.275	0.529
	3.8		0.563	0.399	290	0.082	0.185	0.500	0.907	0.159
	3.8		0.563	0.399	300	0.144	0.196	0.409	0.758	0.097
	3.8		0.563	0.399	310	0.088	0.085	0.018	0.308	0.325
	3.8		0.563	0.399	320	0.561	0.605	0.615	0.386	0.793

mid-latitudes winter	0.8		0.913	0.856	290	0.778	0.904	0.213	0.914	0.818
	0.8		0.913	0.856	300	0.606	0.761	0.116	0.656	0.908
	0.8		0.913	0.856	310	0.394	0.578	0.024	0.360	0.953
	0.8		0.913	0.856	320	0.151	0.364	0.197	0.037	0.965
	2.3		0.766	0.641	290	0.667	0.809	0.021	**1.011**	0.493
	2.3		0.766	0.641	300	0.566	0.738	0.028	0.849	0.618
	2.3		0.766	0.641	310	0.338	0.540	0.203	0.561	0.620
	2.3		0.766	0.641	320	0.009	0.242	0.479	0.173	0.520
	3.8		0.563	0.399	290	0.361	0.495	0.405	0.872	0.164
	3.8		0.563	0.399	300	0.241	0.405	0.499	0.713	0.101
	3.8		0.563	0.399	310	0.182	0.014	0.891	0.251	0.329
	3.8		0.563	0.399	320	0.847	0.619	**1.525**	0.453	0.792

Mean Error						0.597	0.584	0.453	0.606	0.609

STD Error						0.407	0.282	0.365	0.309	0.293

**Table 4.** Verify the accuracy of the study with ground site data. Note: the NDVI is calculated from VIIRS M5 and M7 channel of this swath, with the expression of NDVI = (M7 − M5)/(M7 + M5).

**Land Type**	**Describe**	**Longitude**	**Latitude**	**NDVI**	***T_g_*/K**	***T_t_*/K**	**Δ*T_g_*_–_*_t_*/K**
Water	West Lake	120.23	31.40	−0.33	295.65	296.09	−0.44
Crop1	Wheatland	114.35	32.97	0.59	305.65	304.65	1.00
Crop2	Wheatland	117.97	36.89	0.61	307.25	307.24	0.01
Crop3	Wheatland	117.19	37.18	0.59	305.35	304.27	1.08
City1	Shangqiu	115.67	34.45	0.18	309.05	310.52	−1.47
City2	Shanghai	121.43	31.20	0.25	309.55	308.93	0.62
City3	Hangzhou	120.17	30.23	0.33	313.75	314.15	−0.40

**Table 5.** Retrieval accuracy comparison between VIIRS NOAA LST product and this study.

**Land Type**	**Bands BT/K**		**Our**		**NOAA**		**Δ*_M_*_15_**		**Δ*_M_*_16_**	

	**M15**	**M16**	**M15**	**M16**	**M15**	**M16**	**Our**	**NOAA**	**Our**	**NOAA**	
Water	291.93	291.90	291.82	292.02	290.85	291.18	−0.11	−1.08	0.12	−0.72
City	310.85	310.86	310.68	310.59	309.55	309.49	−0.17	−1.29	−0.27	−1.37
Crop1_0.68_	299.93	299.74	299.24	299.10	298.50	297.66	−0.85	−1.43	−1.54	−2.08
Crop2_0.68_	299.93	299.74	299.24	299.10	299.71	299.54	−0.69	−0.22	−0.64	−0.2
Crop1_0.30_	303.14	302.89	302.19	301.14	301.46	300.47	−0.95	−1.68	−1.75	−2.42
Crop2_0.30_	303.14	302.89	303.13	303.41	302.09	302.42	−0.01	−1.05	0.52	−0.47
Mean1 Error							−0.52	−1.37	−0.86	−1.65
Mean2 Error							−0.25	−0.91	−0.07	−0.69

**Table 6.** Retrieval parameters of input data.

**Land Type**	**Pixel Number**	**Our**			**NOAA**			**Vapor g/cm^−2^**

		***ε*_15_**	***ε*_16_**	**LST/K**	***ε*_15_**	***ε*_16_**	**LST/K**	
Water	91	0.990	0.990	292.46	0.990	0.990	291.22	2.29
City	758	0.974	0.979	313.15	0.974	0.979	311.96	0.70
Crop NDVI = 0.68	929	0.964(0.990)	0.959(0.990)	302.01(300.82)	0.964(0.990)	0.959(0.990)	301.35	1.39
Crop NDVI = 0.30	631	0.964(0.974)	0.959(0.981)	305.41(305.76)	0.964(0.967)	0.959(0.977)	304.60	1.29
